# Understanding Health in Spanish Children: The Role of Demographics in Physical Activity and Nutrition Attitudes

**DOI:** 10.3390/children12070811

**Published:** 2025-06-20

**Authors:** Alvaro Pano-Rodriguez, Carme Jove Deltell, Vicenç Hernández-González, Rafel Cirer-Sastre, Alejandro Legaz-Arrese, Joaquin Reverter-Masia

**Affiliations:** 1 Department of Education Science, Faculty of Education, Psychology and Social Work, University of Lleida, 25003 Lleida, Spain; alvaro.depano@udl.cat (A.P.-R.); carme.jove@udl.cat (C.J.D.); joaquin.reverter@udl.cat (J.R.-M.); 2 Consolidated Research Group Human Movement Generalitat de Catalunya, University of Lleida, 25003 Lleida, Spain; rcirer@gencat.cat; 3 National Institute of Physical Education of Catalonia, University of Lleida, 25192 Lleida, Spain; 4 Section of Physical Education and Sports, Faculty of Health and Sport Sciences, University of Zaragoza, 22002 Huesca, Spain; alegaz@unizar.es

**Keywords:** health promotion, lifestyle factors, self-reported fitness, eating habits, childhood development, demographic disparities, survey research

## Abstract

**Background**: This study investigates the influence of sex, age, and their interaction on health behaviors, perceived physical fitness, and attitudes toward physical activity and eating among schoolchildren. **Methods**: A cross-sectional analysis was conducted on 1027 students aged 8–12 years from 15 primary schools in Lleida. Participants completed the PAQ-A for physical activity, the IFIS for perceived physical fitness, the AATPA for attitudes toward physical activity, and the AATE for attitudes toward eating. Data were analyzed using regression models to assess the effects of sex, age, and their interaction. **Results**: Males reported higher physical activity levels and more positive attitudes toward physical activity than females (*p* < 0.05). Perceived physical fitness was also greater among males (*p* < 0.05). Age was positively associated with physical activity and attitudes toward healthy eating, with older children reporting more favorable outcomes (*p* < 0.01). Significant interaction effects between sex and age were observed for physical activity levels and attitudes toward eating, indicating demographic-specific trends. **Conclusions**: This study highlights significant sex and age differences in children’s health behaviors and attitudes. These findings underscore the need for gender-sensitive and age-appropriate interventions to promote healthier lifestyles among schoolchildren. Future research should explore longitudinal designs to confirm these relationships over time.

## 1. Introduction

Children’s health behaviors are crucial in shaping long-term health outcomes, influencing the prevalence of chronic diseases, mental health conditions, and overall well-being. Key behavioral indicators such as physical activity levels, perceived physical fitness, and attitudes toward physical activity and healthy eating are particularly relevant during the early school years when foundational habits are formed [1,2]. Understanding these behaviors during adolescence is essential for developing interventions aimed at promoting healthier lifestyles and reducing future health risks [3,4,5].

Evaluating children’s health behaviors is imperative for designing effective public health policies and individual interventions. The current literature highlights the complexity of these behaviors, which are influenced by socioeconomic status, cultural norms, psychological factors, and biological determinants [6,7]. By examining these behaviors through validated variables, researchers can identify key areas for intervention and monitor changes over time. The academic value of this research lies in its potential to fill gaps in the existing literature by providing updated, thorough data on health behavior determinants among children, thereby supporting evidence-based public health strategies.

In recent decades, the exploration of children’s health behaviors has been strengthened by advances in behavioral science and epidemiology, especially through large-scale quantitative studies. For example, a 2021 systematic review demonstrated that nature contact significantly benefits physical activity and mental health in children aged 5–12 years [8]. Moreover, research from 2022 to 2023 has highlighted how sociodemographic factors—such as age, gender, or socioeconomic context—shape children’s health behaviors during and after challenging events such as the COVID-19 pandemic [9]. These studies reflect contemporary methodological sophistication and substantiate the relevance of our focus on demographic determinants. Comprehensive reviews and meta-analyses have also highlighted the multifaceted nature of adolescent health behaviors and their significant impact on long-term health outcomes [10,11]. For instance, regular physical activity during adolescence is linked to lower risks of obesity and cardiovascular disease, while poor dietary habits are associated with increased risks of chronic diseases later in life [12,13]. Additionally, perceived physical fitness and positive attitudes toward physical activity and healthy eating play vital roles in adopting and maintaining healthy behaviors [14,15].

Despite international recommendations promoting daily physical activity and a balanced diet for children, large proportions of the population fail to meet these standards [16]. Numerous studies have pointed out that sex and age are consistent determinants of variability in these behaviors. For instance, girls are often less physically active than boys, and this disparity tends to increase with age [17,18].

Perceptions of physical fitness also differ by gender. Boys typically report higher perceived physical fitness, which may reflect both social expectations and actual differences in physical activity involvement [1]. These self-perceptions are important predictors of motivation, self-efficacy, and continued participation in physical activity throughout life [2].

Attitudinal differences have also been documented. Recent reviews suggest that girls tend to show more positive attitudes toward healthy eating, while boys display greater enthusiasm for physical activity [18,19]. These differences are shaped by social, cultural, and environmental factors and highlight the need for tailored health interventions.

Although the relationship between sex, age, and health-related behaviors has been studied internationally, most research has focused on adolescents or on individual outcomes rather than a combined perspective in school-aged children. Furthermore, there is a lack of studies within the Spanish context that simultaneously assess objective and subjective dimensions of health behaviors—such as physical activity, fitness perception, and attitudes—across a large and representative population of primary schoolchildren.

In Spain, schools serve as a critical setting for health promotion. However, there is limited empirical evidence based on integrated behavioral models and validated tools to guide the design of demographic-sensitive interventions. Addressing this gap is essential to develop and implement strategies that are culturally appropriate and grounded in current local data.

Therefore, this study aims to examine how sex and age are associated with physical activity, perceived fitness, and nutrition attitudes in Spanish primary schoolchildren. This multifaceted analysis helps identify trends and disparities that are relevant for the design of future school- and community-based health interventions.

We hypothesize that boys will report higher physical activity levels and perceived physical fitness than girls, whereas girls will show more positive attitudes toward healthy eating. We also expect age-related trends, with older children presenting more favorable attitudes and behaviors, and interaction effects that reflect diverging patterns between boys and girls over time.

## 2. Materials and Methods

### 2.1. Study Design

This study employs a cross-sectional design to investigate the physical activity level, perceived physical fitness, and attitudes toward physical activity and eating among children. The cross-sectional approach has followed the instructions of the STROBE Statement for reporting observational studies. This study adhered to the latest Declaration of Helsinki, dated October 2013.

### 2.2. Ethics and Consent

This research was approved by the Ethics Committee for Clinical Research of the Catalan Sports Council, with a favorable assessment issued on 15 December 2020 (Project No. 30/CEICGC/2020).

### 2.3. Participants

The study population comprised schoolchildren born between 2010 and 2014, who were enrolled in 3rd through 6th grades during the 2021–2022 academic year in the province of Lleida. Data collection took place between November and December 2021. A total of 1455 students were initially invited to participate. To ensure age-appropriate comprehension of the questionnaires, only students in the intermediate and upper levels of primary education were included in this analysis. Initially, the questionnaires were administered to a total of 1396 students. Upon reviewing the responses, 325 students from the 1st and 2nd grades of primary school were excluded, as the instruments used were deemed less reliable for this younger age group. In addition, 44 responses were excluded due to incomplete data or inconsistent answers. Consequently, after data cleaning, the final sample consisted of 1027 students from the middle and upper stages of primary education (3rd, 4th, 5th, and 6th grades) of whom 470 were boys (46%) and 557 girls (54%). This final sample was confirmed in coordination with the co-author responsible for the statistical analyses. In line with the exploratory nature of this study, we did not conduct a formal a priori power analysis. This study aimed to describe sex- and age-related patterns in physical activity, perceived fitness, and health-related attitudes, rather than to test specific theoretical models or predefined effect sizes. For this reason, we prioritized enrolling the maximum number of eligible students across the participating schools during the data collection period. The resulting sample of 1027 participants is considered substantial and suitable for the multivariate analyses conducted.

Inclusion criteria required that participants be enrolled in school, within the specified age range, and capable of providing written informed consent, with parental consent obtained where applicable. This consent included permission to publish clinical data. Exclusion criteria encompassed any condition, reported by parents or teachers, that might interfere with the completion or interpretation of the questionnaires.

Initial communication between the research team, teachers, school administration, families, and students was given vital importance. Meetings with the Parents’ Associations (AMPAS) were fundamental from the beginning to effectively convey the methodology for collecting information through the evaluation tools used to assess the variables. A total of 26 members were part of the working group, including researchers, teachers, and monitors, ensuring comprehensive collaboration and support throughout the study.

### 2.4. Outcomes

This study assessed several key variables related to children’s health behaviors, perceived physical fitness, and attitudes toward physical activity and eating. The following sections detail these variables and the specific assessment tools used for each.

#### 2.4.1. Physical Activity Level

Physical activity levels were measured using the Physical Activity Questionnaire for Adolescents (PAQ-A). The PAQ-A is a self-administered, 7-day recall questionnaire designed to provide a general estimate of physical activity levels, focusing on participation in different physical activities, as well as activity during physical education classes, lunch breaks, after school, evenings, and weekends. It comprises several items scored on a 5-point Likert scale, with higher scores indicating higher levels of physical activity. The PAQ-A is a well-validated tool for assessing physical activity in younger populations [20]. For this study, a composite score was obtained by averaging responses to questions 2 to 7. The PAQ-A has demonstrated robust psychometric properties in various studies.

#### 2.4.2. Perceived Physical Fitness

Perceived physical fitness was assessed using the International Fitness Scale (IFIS). The IFIS is a self-reported measure that encompasses dimensions such as cardiorespiratory fitness, muscular strength, speed, and flexibility. Participants rate their fitness on a 5-point scale relative to their peers. Composite scores for the IFIS were obtained by averaging all the questions. The IFIS has been validated in several international studies, confirming its reliability and relevance for younger populations [21].

#### 2.4.3. Attitudes Toward Physical Activity

The Adolescent Attitudes Toward Physical Activity (AATPA) questionnaire was used to evaluate specific attitudes toward various forms of physical activity, including team sports, individual sports, and recreational activities. This 12-item instrument uses a 5-point Likert scale to capture a range of attitudes, with higher scores reflecting more favorable attitudes toward physical activity. Items 3, 5, 6, and 7 of the AATPA questionnaire were scored on a reverse scale, where healthier behaviors were denoted by the lowest scores. Before analysis, scores of those items were reversed to allow direct comparisons and averaging with the rest of the items. Composite scores for the AATPA were obtained by averaging all the questions. The AATPA has demonstrated good internal consistency and construct validity in multiple settings. Its design and validation have been thoroughly documented, confirming its suitability for measuring attitudes toward physical activity in young individuals [22].

#### 2.4.4. Attitudes Toward Eating

Attitudes toward eating were assessed using the Adolescent Attitudes Toward Eating (AATE) questionnaire. The AATE focuses on adolescents’ attitudes toward healthy eating, perceived barriers to maintaining a healthy diet, and the influence of social and environmental factors on eating behaviors. It includes a series of items rated on a 5-point Likert scale, with higher scores indicating more positive attitudes toward healthy eating. Items 4, 6, 7, 8, and 9 of the AATE questionnaire were scored on a reverse scale, where healthier behaviors were denoted by the lowest scores. Before analysis, scores of those items were reversed to allow direct comparisons and averaging with the rest of the items. Composite scores for the AATE were obtained by averaging all the questions. This tool was designed and validated to capture the complexities of dietary attitudes in younger populations, ensuring its reliability and validity [22].

#### 2.4.5. Sociodemographic Variables

Key sociodemographic variables, including age, sex, and socioeconomic status, were collected through a demographic survey. These variables were used to control for potential confounding factors and to analyze their interactions with health behaviors and attitudes. Socioeconomic status was assessed using a composite index based on parental education, occupation, and household income.

By utilizing these well-validated assessment tools, this study aims to provide a comprehensive analysis of health behaviors, perceived physical fitness, and attitudes toward physical activity and eating among children. The data collected through these instruments were analyzed to explore the relationships and interactions between various health-related variables and demographic factors.

### 2.5. Statistical Analysis

Continuous data were normally distributed and described with their mean and standard deviation from the mean. On the other hand, the scores obtained from the Likert scales were considered ordinal categorical variables and summarized with the absolute and relative frequencies in each category. The effects of sex, age, and their interaction were assessed using regression models and their respective ANOVAs. To this end, continuous variables were modeled using general linear models, whereas ordinal discrete variables were modeled using ordered logistic or probit regression [23]. In addition, a matrix of correlations between items was calculated using Spearman coefficients of correlation (p). The complete details of the coefficients, their 95% confidence intervals, and *p*-values are provided in the Supplementary Materials, File S1.

## 3. Results

### 3.1. Descriptive Characteristics of the Sample

Table 1 presents the descriptive statistics of the participants by sex and grade level. The final sample included 1027 students, with 470 boys (46%) and 557 girls (54%). The mean age was 9.84 years (SD = 1.19). Students were distributed across 3rd to 6th grades, with the largest group in 6th grade (27%) and the smallest in 3rd grade (22%).

### 3.2. Physical Activity Levels

**Figure 1:**
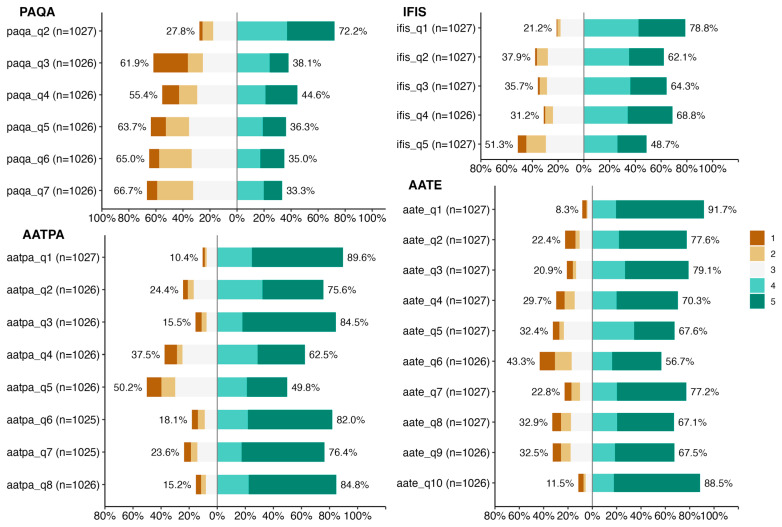
Overall results of Likert scores by questionnaire: PAQ-A (Physical Activity Questionnaire for Adolescents), IFIS (International Fitness Scale), AATPA (Adolescent Attitudes Toward Physical Activity), and AATE (Adolescent Attitudes Toward Eating). Each horizontal bar represents the percentage of participants that selected each response option on a 5-point Likert scale (1 = strongly disagree, 5 = strongly agree). The total number of valid responses for each item is shown in parentheses.

The PAQ-A scores were averaged to create a composite score for physical activity levels. The average PAQ-A score for the sample was 3.2 ± 0.9, indicating moderate physical activity levels across the cohort. Significant effects were found for sex, with males reporting higher levels of physical activity than females (*p* = 0.008). Age also had a significant effect (*p* = 0.014), indicating that older children tended to report higher physical activity levels. The interaction between sex and age was significant (*p* = 0.005), suggesting that the effect of age on physical activity levels differed between males and females (Table 2, Figure 1).

### 3.3. Perceived Physical Fitness

**Figure 2:**
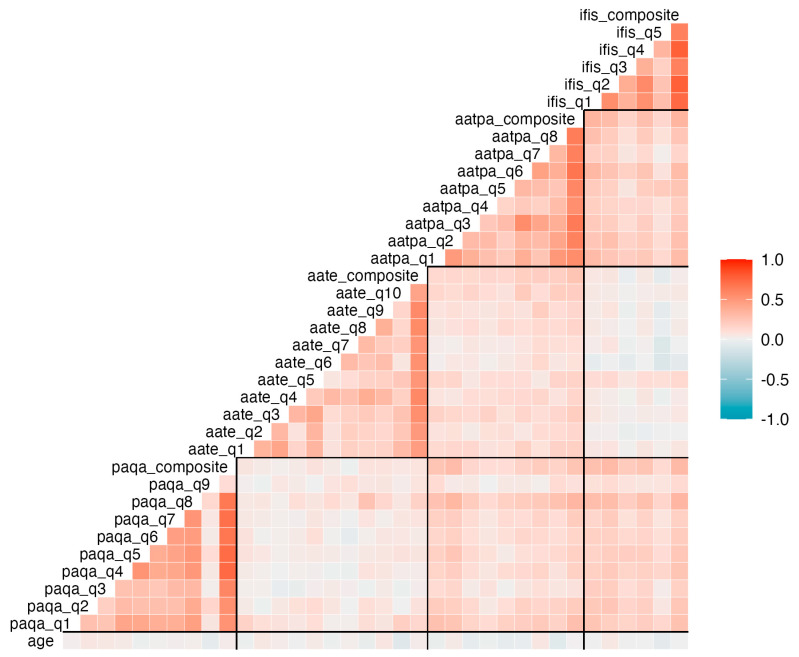
Heat map of the correlation matrix among items. The figure shows Spearman correlation coefficients between age and individual items, as well as composite scores from the PAQ-A (Physical Activity Questionnaire for Adolescents), IFIS (International Fitness Scale), AATPA (Adolescent Attitudes Toward Physical Activity), and AATE (Adolescent Attitudes Toward Eating) questionnaires. Each square represents the strength and direction of the correlation (red = positive, blue = negative; intensity indicates magnitude). Composite scores are computed as the mean of corresponding item scores.

The IFIS scores showed that most children rated their physical fitness as average to above average, with a mean score of 3.6 ± 0.8. This suggests a generally positive perception of physical fitness among the participants. The analysis revealed a significant effect of sex for ifis_q3 (*p* = 0.028), with males generally reporting higher fitness levels. There were no significant effects of age or interactions between sex and age for the other IFIS items (Table 2, Figure 2).

### 3.4. Attitudes Toward Physical Activity

The AATPA questionnaire revealed favorable attitudes toward physical activity, with an average score of 4.1 ± 0.7. Higher scores were consistently observed among males compared to females, indicating a more positive attitude toward physical activity among male adolescents. Significant differences between males and females were observed for aatpa_q2 (*p* = 0.048) and aatpa_q8 (*p* = 0.033). No significant effects of age or interactions were found for other AATPA items (Table 2, Figure 1).

### 3.5. Attitudes Toward Eating

The AATE scores indicated positive attitudes toward healthy eating, with a mean score of 4.0 ± 0.8. Female participants generally reported higher scores than males, suggesting a more positive attitude toward healthy eating among female children. Age had significant effects on aate_q2 (*p* < 0.001), aate_q5 (*p* = 0.007), and aate_q10 (*p* = 0.003), indicating that older children generally had more positive attitudes toward healthy eating. Significant interaction effects between sex and age were found for aate_q2 (*p* = 0.014) and aate_q5 (*p* = 0.009) (Table 2, Figure 2).

## 4. Discussion

This study examined the effects of sex, age, and their interaction on health behaviors, perceived physical fitness, and attitudes toward physical activity and eating in a representative sample of Spanish primary schoolchildren. The results showed that boys reported higher levels of physical activity and perceived fitness than girls, while older children showed lower physical activity and less positive attitudes toward healthy eating. Significant associations were observed between age, sex, and all the studied health behaviors.

These findings align with previous research indicating that boys are generally more physically active than girls [16,17]. For instance, a cross-sectional accelerometry study in 8–9-year-old children from Andalusia reported that boys accumulated significantly more vigorous physical activity (19.9 min/day) compared to girls (11.4 min/day) [24]. Similarly, in a sample of 455 nine-year-old children from Spanish schools, boys also showed significantly higher overall physical activity levels than girls (*p* ≤ 0.001) [25]. Our results are also consistent with the previous literature, showing that girls tend to exhibit more positive attitudes toward healthy eating [18,19]. However, our study offers unique insights by highlighting interaction effects between sex and age, particularly in physical activity levels and attitudes toward eating. Our observations suggest that demographic influences on health behaviors are dynamic and evolve throughout childhood.

While many prior studies have analyzed the influence of sex or age separately, few have examined their combined effects on children under 13. Our findings show, for instance, that the gap in physical activity between boys and girls widens with age, which may reflect differences in socialization, confidence, or access to physical activity opportunities [1,2]. These findings underscore the need for targeted strategies in schools and communities to promote physical activity among girls as they age, ensuring equitable opportunities and support. In this sense, gender-differentiated early interventions may be key to preventing the widening of these disparities over time.

Similarly, attitudes toward healthy eating improved with age, especially among girls, supporting the idea that health-related attitudes are shaped progressively and can be positively influenced by early education efforts.

Importantly, this study contributes context-specific data from a Spanish population, addressing a gap in the literature. By simultaneously analyzing physical activity, fitness perception, and attitudes using validated instruments, we offer a more integrated understanding of how health behaviors manifest and interact during the school years. This comprehensive approach enhances the applicability of our findings to the development of public health and school-based interventions that are both age-appropriate and gender-sensitive. These findings broadly support our initial hypothesis. Specifically, the results confirmed that boys report higher physical activity levels and perceived physical fitness, while girls show more positive attitudes toward healthy eating. The data also revealed significant age-related trends, with older children generally reporting healthier behaviors and attitudes. Moreover, the presence of interaction effects between sex and age—particularly in physical activity levels and attitudes toward eating—underscores the importance of examining demographic factors in combination rather than in isolation. These patterns suggest that behavioral differences are not static across childhood but evolve as children grow older, reinforcing the need for early, tailored interventions.

The significant interaction between sex and age suggests that the gap in physical activity levels between boys and girls widens as they grow older. This could be due to the different socialization processes experienced by boys and girls, where boys might receive more encouragement and opportunities to engage in physical activities [26]. Additionally, the decline in physical activity observed in girls could be influenced by cultural factors, body image concerns, and a lack of interest or confidence in physical activities [27].

This study also revealed that males generally reported higher levels of perceived physical fitness compared to females. This finding is consistent with previous research suggesting that boys often perceive themselves as being more physically fit than girls, which could be influenced by gender stereotypes and societal expectations [28]. The absence of significant age-related differences in perceived physical fitness suggests that these perceptions are established early in adolescence and are less likely to fluctuate with age [29]. This study also revealed that males generally reported higher levels of perceived physical fitness. Perceptions of physical fitness play a key role in boosting self-efficacy, which is a strong predictor of motivation and adherence to health-promoting behaviors, including structured physical activity programs [30]. Individuals with higher self-efficacy are more likely to initiate and maintain regular physical activity, suggesting that the observed gender differences in fitness perception could influence how boys and girls engage with—and benefit from—school or community health interventions.

The higher self-reported fitness levels among boys could also reflect greater engagement in vigorous physical activities, which are more likely to be valued and encouraged in male-dominated sports and physical activities [31]. This gender disparity in perceived fitness might contribute to differences in motivation and self-efficacy related to physical activity, potentially influencing long-term participation in physical activities [32].

The results showed that boys exhibited more favorable attitudes toward physical activity than girls. This aligns with prior research indicating that boys generally have more positive attitudes toward sports and physical exercise, which is often reflected in higher participation rates [16,17,33]. These attitudes are crucial as they can influence the likelihood of engaging in and maintaining physical activity over time [34]. The findings suggest that boys’ more positive attitudes may stem from societal norms that associate masculinity with athleticism and physical competence, whereas girls may encounter more societal barriers and less encouragement to participate in physical activities [14,35].

The lack of significant age effects on attitudes toward physical activity implies that these attitudes are relatively stable throughout adolescence. This stability highlights the importance of early interventions to foster positive attitudes toward physical activity, particularly among girls [2]. Programs designed to increase girls’ confidence in their physical abilities and provide positive experiences in physical activity settings could help mitigate these disparities [1,36]. These attitudes are shaped not only by individual preferences but also by broader sociocultural factors. For instance, gender norms, parental modeling, and media exposure significantly influence children’s perceptions of physical activity and healthy eating [37,38]. Moreover, parents’ dietary habits and physical activity behaviors at home have been shown to impact children’s lifestyle patterns [39].

Interestingly, this study revealed that girls reported more positive attitudes toward healthy eating compared to boys, and these attitudes tended to become more favorable with age. This finding is consistent with previous research suggesting that girls are more likely to be concerned with health and body image, leading them to adopt healthier eating habits [40,41]. The positive correlation between age and attitudes toward healthy eating may reflect increased awareness and understanding of the importance of nutrition as children grow older and gain more autonomy over their food choices.

The significant interaction effects between sex and age on attitudes toward eating indicate that while both boys and girls may become more health-conscious with age, the rate of change may differ. Girls might experience a more pronounced shift toward positive attitudes due to greater societal emphasis on appearance and health consciousness among females [18,41]. These findings underscore the need for tailored nutritional education programs that address the specific needs and perceptions of boys and girls, potentially helping to bridge the gap in attitudes toward healthy eating.

### 4.1. Implications for Public Health Interventions

The significant gender differences observed in physical activity levels and attitudes toward physical activity and eating suggest that public health interventions must be gender-sensitive and culturally appropriate. For example, programs aiming to increase physical activity among girls should focus on activities that are appealing and accessible to them, address perceived barriers, and provide positive role models [27]. Similarly, nutritional interventions should consider the different motivations and challenges faced by boys and girls, potentially incorporating elements that address body image concerns and promote a positive relationship with food [19].

Early, school-based interventions that actively engage students in physical activity and nutrition education are particularly promising. Integrating structured physical activity sessions, participatory cooking workshops, and curriculum-linked health modules may enhance both motivation and knowledge. Additionally, family involvement programs—such as joint activity planning and nutrition challenges—can reinforce healthy behaviors at home [42]. Tailoring these interventions by age and gender could improve their effectiveness and long-term adherence.

The positive association between age and both physical activity levels and attitudes toward healthy eating underscores the importance of sustained health education throughout adolescence. School-based programs that integrate comprehensive physical education and nutrition education can play a critical role in reinforcing healthy behaviors. Additionally, engaging parents and communities in these programs can help create supportive environments that foster healthy lifestyle choices [43].

### 4.2. Limitations and Future Research

While this study provides valuable insights, several limitations should be noted. The cross-sectional design precludes causal inferences, and the reliance on self-reported data may introduce bias. Future research should employ longitudinal designs to better understand the causal relationships and dynamics of these health behaviors and attitudes over time. Moreover, qualitative studies could provide deeper insights into the underlying motivations and barriers influencing children’s health behaviors, offering a more nuanced understanding of the observed gender differences.

## 5. Conclusions

This study aimed to analyze how sex and age are associated with physical activity, perceived physical fitness, and attitudes toward healthy eating in Spanish primary schoolchildren. The findings reveal that boys generally report higher levels of physical activity and perceived fitness than girls, while girls exhibit more favorable attitudes toward healthy eating. Moreover, older children showed lower levels of physical activity and less positive attitudes compared to younger peers.

Significant interaction effects between sex and age were also observed, indicating that these demographic influences are not fixed but evolve throughout childhood. These results underscore the need for health promotion strategies that are sensitive to both age and gender and support the implementation of early, tailored interventions within schools and families.

## Figures and Tables

**Table 1:** Descriptive statistics of participants by sex and grade level.

Variable	M (SD)/n (%)
Sex	
Male	470 (46%)
Female	557 (54%)
Age (years)	9.84 (1.19)
Grade	
3rd	221 (22%)
4th	265 (26%)
5th	261 (25%)
6th	280 (27%)

M = mean; SD = standard deviation; and n = number of participants. Age is reported as mean (standard deviation); categorical variables are reported as counts and percentages.

**Table 2:** Sex and age differences across items.

Item	Likert Scores (1–5) ^1^										*p*-Values ^2^		
	1		2		3		4		5		Sex	Age	Interaction
Physical Activity Questionnaire for Adolescents (PAQ-A)													
paqa_q2	24	(2.3%)	81	(7.9%)	181	(17.6%)	380	(37%)	361	(35.2%)	0.065	**0.014**	**0.005**
paqa_q3	262	(25.5%)	114	(11.1%)	259	(25.2%)	248	(24.1%)	143	(13.9%)	**0.008**	**0.006**	0.11
paqa_q4	128	(12.5%)	138	(13.4%)	302	(29.4%)	216	(21%)	242	(23.6%)	0.29	**0.003**	0.41
paqa_q5	114	(11.1%)	176	(17.1%)	364	(35.4%)	196	(19.1%)	176	(17.1%)	0.85	**0.038**	0.148
paqa_q6	76	(7.4%)	247	(24.1%)	344	(33.5%)	177	(17.2%)	182	(17.7%)	0.27	0.20	0.084
paqa_q7	78	(7.6%)	273	(26.6%)	333	(32.4%)	203	(19.8%)	139	(13.5%)	0.38	0.09	0.22
International Fitness Scale (IFIS)													
ifis_q1	7	(0.7%)	25	(2.4%)	186	(18.1%)	436	(42.5%)	373	(36.3%)	0.32	0.62	0.19
ifis_q2	15	(1.5%)	87	(8.5%)	287	(27.9%)	361	(35.2%)	277	(27%)	0.64	0.82	0.57
ifis_q3	15	(1.5%)	58	(5.6%)	294	(28.6%)	371	(36.1%)	289	(28.1%)	**0.028**	0.087	0.085
ifis_q4	12	(1.2%)	62	(6%)	246	(24%)	350	(34.1%)	356	(34.7%)	0.17	0.74	0.95
ifis_q5	68	(6.6%)	156	(15.2%)	303	(29.5%)	268	(26.1%)	232	(22.6%)	0.75	0.45	0.73
Adolescent Attitudes Toward Physical Activity (AATPA)													
aatpa_q1	16	(1.6%)	14	(1.4%)	77	(7.5%)	254	(24.7%)	666	(64.8%)	0.62	0.34	0.27
aatpa_q2	35	(3.4%)	43	(4.2%)	172	(16.7%)	331	(32.2%)	445	(43.3%)	**0.048**	0.55	0.20
aatpa_q3	45	(4.4%)	36	(3.5%)	78	(7.6%)	184	(17.9%)	683	(66.5%)	0.67	0.81	0.66
aatpa_q4	91	(8.9%)	40	(3.9%)	254	(24.7%)	295	(28.7%)	346	(33.7%)	0.87	0.48	0.15
aatpa_q5	107	(10.4%)	101	(9.8%)	307	(29.9%)	217	(21.1%)	294	(28.6%)	0.59	0.89	0.74
aatpa_q6	44	(4.3%)	50	(4.9%)	91	(8.9%)	224	(21.8%)	616	(60%)	0.34	0.28	0.77
aatpa_q7	50	(4.9%)	47	(4.6%)	145	(14.1%)	178	(17.3%)	605	(58.9%)	0.14	**0.001**	0.35
aatpa_q8	38	(3.7%)	35	(3.4%)	83	(8.1%)	230	(22.4%)	640	(62.3%)	**0.033**	0.857	0.29
Adolescent Attitudes Toward Eating (AATE)													
aate_q1	36	(3.5%)	8	(0.8%)	41	(4%)	200	(19.5%)	742	(72.2%)	0.54	0.46	0.12
aate_q2	88	(8.6%)	35	(3.4%)	107	(10.4%)	225	(21.9%)	572	(55.7%)	0.40	**<0.001**	**0.014**
aate_q3	51	(5%)	27	(2.6%)	137	(13.3%)	276	(26.9%)	536	(52.2%)	0.61	0.45	0.38
aate_q4	71	(6.9%)	85	(8.3%)	149	(14.5%)	205	(20%)	517	(50.3%)	0.61	0.96	0.9
aate_q5	55	(5.4%)	38	(3.7%)	240	(23.4%)	353	(34.4%)	341	(33.2%)	0.21	**0.007**	**0.009**
aate_q6	129	(12.6%)	141	(13.7%)	174	(16.9%)	167	(16.3%)	415	(40.4%)	0.85	0.52	0.93
aate_q7	58	(5.6%)	72	(7%)	104	(10.1%)	208	(20.3%)	585	(57%)	0.99	0.41	0.37
aate_q8	74	(7.2%)	84	(8.2%)	180	(17.5%)	209	(20.4%)	480	(46.7%)	0.74	0.62	0.74
aate_q9	70	(6.8%)	81	(7.9%)	183	(17.8%)	191	(18.6%)	501	(48.8%)	0.80	**0.016**	0.77
aate_q10	44	(4.3%)	18	(1.8%)	56	(5.5%)	182	(17.7%)	726	(70.7%)	0.28	**0.003**	0.91

Overall results of Likert scores by questionnaire, with age and group comparisons at item level: PAQ-A = Physical Activity Questionnaire for Adolescents; IFIS = International Fitness Scale; AATPA = Adolescent Attitudes Toward Physical Activity; and AATE = Adolescent Attitudes Toward Eating. ^1^ Likert scores are treated as ordinal categories and have been summarized with absolute (relative) frequencies. ^2^ *p*-values refer to the ANOVA main effects obtained from item-level ordinal logistic regressions. Bolded *p*-values indicate statistically significant differences (*p* < 0.05).

## Data Availability

The datasets generated during and/or analyzed during the current study are available from the corresponding author upon reasonable request due to ethical restrictions related to the protection of sensitive information and privacy of underage participants, in accordance with institutional and data protection regulations.

## Supplementary Materials

The following supporting information can be downloaded at: https://www.mdpi.com/article/10.3390/children12070811/s1.
